# Clinical reasoning for performance of transesophageal echocardiography in veterans with *Staphylococcus aureus* bacteremia

**DOI:** 10.1017/ash.2023.493

**Published:** 2023-12-04

**Authors:** Emily C. Woods, Trisha S. M. Nakasone, Cybele A. Renault

**Affiliations:** 1 Stanford University School of Medicine, Stanford, CA, USA; 2 Veterans Affairs Palo Alto Health Care System, Palo Alto, CA, USA

## Abstract

This study examined physicians’ reasoning about obtaining transesophageal echocardiography (TEE) in cases of *Staphylococcus aureus* bacteremia (SAB). In 221 cases of SAB over 5 years, the most common reasons for not performing TEE were clinical response to antibiotics, negative TTE results, and the expectation that TEE would not change management.

## Introduction

*Staphylococcus aureus* causes infective endocarditis (IE) in ∼43,000 people annually in the United States.^1^ Prolonged antibiotic courses needed to treat IE increase the risk of side effects and development of antibiotic resistance.^2,3^ Conversely, a missed diagnosis of IE can lead to significant morbidity and mortality.^4^ Therefore, accurate diagnosis of IE in patients with *Staphylococcus aureus* bacteremia (SAB) is critical.

Diagnosis of IE is classically made using the modified Duke criteria, which includes echocardiographic evidence of cardiac involvement.^5^ Given the high risk for IE in patients with SAB, echocardiography is recommended for all patients with SAB.^6,7^ The choice of which type of echocardiography to obtain can be challenging because transthoracic echocardiograms (TTEs) are noninvasive but lack the sensitivity of transesophageal echocardiograms (TEEs). Several scoring tools, such as VIRSTA,^8^ can help clinicians identify patients who are at particularly high risk for IE and who may therefore need a TEE; however, it is unclear how often clinicians use these tools to guide decisions.

Understanding how such decisions are made can help inform targeted education and institutional guidelines to optimize patient care. To this end, we performed a retrospective review of the clinical reasoning used by physicians regarding performance of TEE in patients with SAB hospitalized at the Palo Alto Veterans Health Care System (VAPAHCS).

## Methods

PraediAlert clinical surveillance software was used to identify all blood cultures positive for *S. aureus* at the 109-acute care bed VAPAHCS hospital between 01/01/2016 and 12/31/2021. Patients were included if they had at least one blood culture positive for *S. aureus* during this time. Patients were excluded if they left VAPAHCS within 48 hours (by transfer or death), had initial work-up performed elsewhere, or were converted to hospice during work-up or treatment.

Clinical and laboratory data for these cases were collected using PraediAlert software and manual chart review. Information obtained from infectious disease (ID) consult notes included: whether a patient had physical examination findings consistent with IE, had sites of infection that physicians considered metastatic, and had complicated or uncomplicated bacteremia,^7^ and whether IE was diagnosed or not. Cases were classified as presumed IE if a definitive diagnosis of IE was not made, but physicians either opted to treat empirically for IE or expressed suspicion for IE but did not pursue definitive diagnostics because the patient required antibiotic treatment for another condition that simultaneously treated IE. ID consult notes were reviewed to assess reasons cited as rationale regarding the decision whether or not to pursue TEE.

Following data collection, we split the data into predetermined subgroups for comparison: patients who underwent TEE compared to those who did not and patients who were diagnosed as having IE compared to those who were not. For comparisons involving means, we calculated the Student two-tailed *t* test (α = 0.05). For comparisons involving categorical variables, we calculated a Fisher’s exact test (α = 0.05).

This work was considered exempt from IRB review per Stanford University Research Compliance Office guidelines.

## Results

We identified 263 cases of SAB at VAPAHCS between 01/01/2016 and 12/31/2021. Forty-two cases met exclusion criteria, leaving 221 cases (84%) in our analysis.

Table 1 shows demographic characteristics, IE risk factors, components of disease course, and aspects of treatment for all cases and for subgroups based on TEE status and IE status.

**Table 1.** Demographics, underlying risk factors, disease characteristics, and treatment course of all analyzed patients and by subgroups (comparing cases in which TEE was performed vs those in which it was not, and cases in which endocarditis was diagnosed vs those in which it was not).

	Number of cases (percentage)				
	All cases	TEE performed	TEE not performed	Endocarditis	Not endocarditis
	(*N* = 221)	(*N* = 46)	(*N* = 175)	(*N* = 39)	(*N* = 182)
Demographics					
Mean age	67.6 ± 11.8	67.5 ± 11.5	67.6 ± 12.0	69.0 ± 13.2	67.3 ± 11.5
Male	215 (97)	45 (98)	170 (97)	39 (100)	176 (97)
Risk factors					
Hemodialysis	22 (10)	9 (20)	13 (7)^b^	4 (10)	18 (10)
ICD/PPM	11 (5)	5 (11)	6 (3)	3 (8)	8 (4)
Prosthetic valve	13 (6)	9 (20)	4 (2)^b^	3 (8)	10 (5)
Valvular disease	19 (9)	9 (20)	10 (6)^b^	5 (13)	14 (8)
Prior history of IE	0 (0)	0 (0)	0 (0)	0 (0)	0 (0)
IVDU	9 (4)	4 (9)	5 (3)	2 (5)	7 (4)
Disease course					
Time to culture clearance, mean ± SD (days)	3.5 ± 3.2	5.1 ± 5.3	3.1 ± 2.1^a^	6.0 ± 5.8	3.0 ± 2.1^a^
Time to defervescence, mean ± SD (days)	1.1 ± 3.4	2.5 ± 7.0	0.8 ± 1.2	2.9 ± 7.6	0.8 ± 1.2
MRSA	87 (39)	17 (37)	70 (40)	15 (38)	72 (40)
Complicated bacteremia^c^	151 (68)	39 (85)	112 (64)^b^	39 (100)	112 (62)^b^
High-grade bacteremia^d^	119 (54)	34 (74)	85 (49)^b^	32 (82)	97 (48)^b^
Infective endocarditis (definitive or presumed)	39 (18)	10 (22)	29 (17)	39 (100)	0 (0)^b^
Concurrent site of infection					
Osteomyelitis	64 (29)	8 (17)	56 (32)	14 (36)	50 (27)
Abscess	30 (14)	7 (15)	23 (13)	10 (26)	20 (11)^b^
Urinary tract infection	14 (6)	3 (7)	11 (6)	0 (0)	14 (8)
Pulmonary	13 (6)	3 (7)	10 (6)	7 (18)	6 (3)^b^
Native joint infection (septic arthritis or bursitis)	11 (5)	4 (9)	7 (4)	5 (13)	6 (3)^b^
Prosthetic joint infection	11 (5)	2 (4)	9 (5)	1 (3)	10 (5)
Cellulitis or other non-abscess SSTI	7 (3)	2 (4)	5 (3)	3 (8)	4 (2)
Septic thrombophlebitis	7 (3)	3 (7)	4 (2)	3 (8)	4 (2)
Endovascular graft	5 (2)	2 (4)	3 (2)	1 (3)	4 (2)
Other	14 (6)	4 (9)	10 (6)	6 (15)	8 (4)^b^
No other site	83 (38)	19 (41)	64 (37)	8 (21)	75 (41)^b^
Treatment					
Time to *S. aureus* coverage, mean ± SD (days)	0.7 ± 1.0	0.5 ± 0.8	0.7 ± 1.1	0.4 ± 0.6	0.8 ± 1.1^a^
Cases that did not cover SAB empirically^e^	47 (21)	9 (20)	38 (22)	3 (8)	44 (24)^b^
Length of treatment, mean ± SD (weeks)	4.9 ± 3.6	5.2 ± 2.5	4.8 ± 3.8	5.8 ± 2.6	4.7 ± 3.7^a^
Cases treated for at least 6 weeks	92 (42)	23 (50)	69 (39)	30 (77)	62 (34)^b^
Time until ID consult, mean ± SD (days)	2.1 ± 1.7	1.7 ± 1.0	2.2 ± 1.8^a^	1.5 ± 1.2	2.2 ± 1.7^a^

Abbreviations: ICD/PPM, implanted cardiac defibrillator/permanent pacemaker; IE, infective endocarditis; IVDU, intravenous drug use; MSSA, methicillin-sensitive *Staphylococcus aureus*; MRSA, methicillin-resistant *S. aureus*; SSTI, skin and soft tissue infection; SAB, *S. aureus* bacteremia; ID, infectious disease.

a *p* < 0.05 by the Student two-tailed *t* test.

b *p* < 0.05 by Fisher’s exact test.

c Complicated bacteremia defined per IDSA guidelines: endocarditis excluded, absence of implanted prostheses, no evidence of metastatic infection, repeat blood cultures negative and defervescence within 72 hours of antibiotics.^7^

d High-grade bacteremia defined as at least three out of four blood culture bottles positive for *S. aureus*.

e Empiric SAB coverage defined as initiation of vancomycin, daptomycin, cefazolin, or nafcillin prior to culture results.

In terms of primary outcomes, TTE was performed in 97% of all cases. The circumstances regarding the seven patients for whom TTE was not performed are detailed in Supplementary Table 1.

Of the patients who underwent TTE, only five patients (2%) had positive findings that were diagnostic of IE. TEE was performed in 21% of all cases. Out of patients who were ultimately diagnosed as having IE, 100% underwent TTE and 30% underwent TEE. Only 20% of patients who were not diagnosed as having IE underwent TEE.

**Figure 1.**
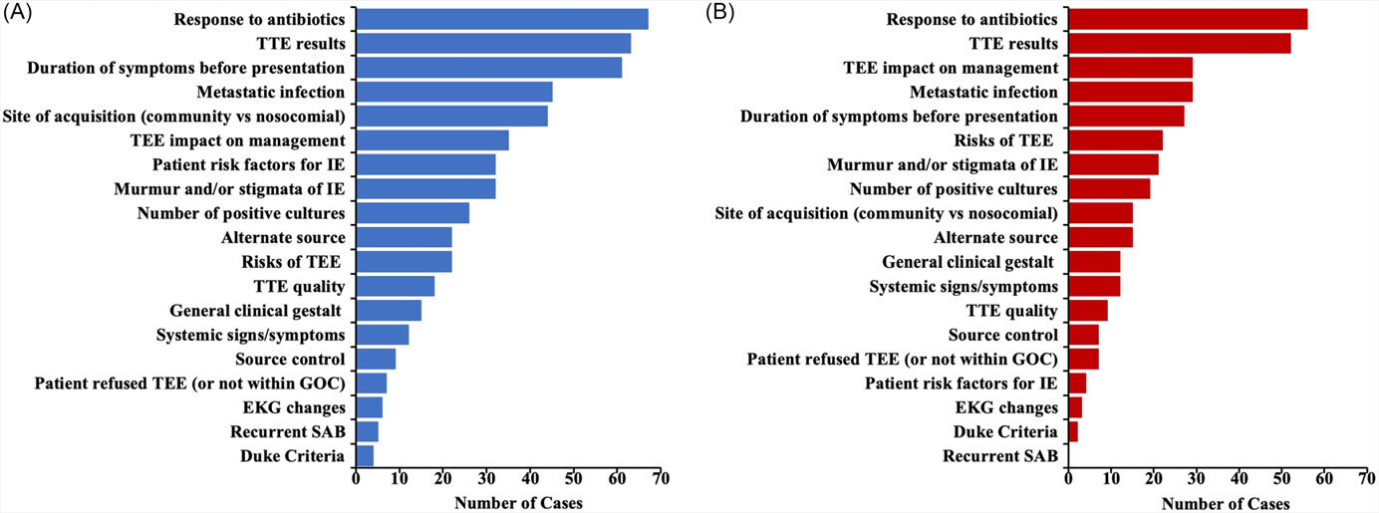
Reasons cited by infectious disease physicians in their recommendations regarding TEE performance. Bar lengths represent the number of cases in which a reason was cited. **(A)** All reasons cited, including those cited in favor of obtaining TEE and those cited as rationale for not obtaining TEE. **(B)** Only reasons that were cited as rationale for not performing TEE.

Our review of ID physician notes revealed 19 recurrent reasons cited in their assessments outlining the reason why TEE should or should not be performed (Figure 1). On average, each case had 2–3 different reasons cited. The 10 most common reasons that physicians cited regarding performance of TEE centered around three main areas: presence/absence of risk factors for IE (personal patient risk factors, duration of symptoms, and site of acquisition), presence/absence of clinical signs concerning for IE (metastatic sites of infection, murmur or other stigmata of IE, response to antibiotics, number of positive blood cultures, and TTE results), and the risk/benefit of performing TEE (patient risks for undergoing TEE and anticipated impact of TEE results on management) (Figure 1A). Among reasons cited for why *not* to obtain TEE, response to antibiotics, TTE results, and impact of TEE on management were the most commonly cited reasons (Figure 1B). In 19 cases (9%), no explicit reasoning regarding TEE performance was mentioned.

## Discussion

Although a number of studies have examined the role of TEE in diagnosis of IE in SAB, to our knowledge, this study is the first to assess ID physicians’ reasoning around the decision to recommend TEE in patients with SAB. By examining physician reasoning, this study advances our understanding of how this often complex diagnostic decision is made in practice.

We found that ID physicians consider a wide range of clinical features and customize their reasoning to each individual case, rather than following a rigid diagnostic algorithm. For example, a predictive scoring tool was explicitly cited in only one case. A CRP value (necessary for calculating the VIRSTA score)^8^ was only measured in 57% of the cases within 7 days of positive blood cultures, indicating that this score was not routinely being calculated. In addition, we found that clinicians were carefully weighing the risks and benefits of TEE for each patient. Using this individualized decision-making did not result in increased rates of adverse events in patients who did not undergo TEE (Supplementary Table 2). Moreover, the rate of IE diagnosed (18%) was similar to rates reported in other studies (7–24%),^9,10^ suggesting that VAPAHCS physicians’ diagnostic reasoning fell within accepted diagnostic practices.

The main limitation of this study is that it is performed at a single clinical site, so the findings may not be applicable to other institutions. In particular, the VAPAHCS population in this study was predominantly over 65 years old, which likely contributed to some reasons, such as risks of TEE, being more commonly cited than they might be in a more age-diverse population. In addition, the older patient population probably contributed to the large number of patients who were ultimately converted to hospice during their treatment course (27 of the 42 excluded patients (10% of all screened cases)).

Nevertheless, this study provides unique insights on physician reasoning around a common diagnostic stewardship question. In an era of escalating healthcare costs and rising awareness of the risks of over-testing, a deeper understanding of physicians’ rationale behind diagnostic test ordering may help inform the design of interventions to optimize appropriate use of TEE in SAB cases. Overall, we found that physicians at VAPAHCS used a variety of clinical features to avoid the overuse of TEE.

## Supporting information

Woods et al. supplementary material Woods et al. supplementary material

## Supplementary material

For supplementary material accompanying this paper visit http://doi.org/10.1017/ash.2023.493. click here to view supplementary material

## Research transparency and reproducibility

Data are available upon request.
