# Supplementary material for: Clinical reasoning for performance of transesophageal echocardiography in veterans with Staphylococcus aureus bacteremia
Source: Antimicrob Steward Healthc Epidemiol. 2023 Dec 4;3(1):e221. doi: 10.1017/ash.2023.493 (PMC10753505; doi:10.1017/ash.2023.493)
Supplement: Woods et al. supplementary material [file S2732494X2300493Xsup001.docx]

Supplementary Tables

**Supplementary Table 1.** Reasons cited by ID for not performing TTE.

| **Reason** | **Number of cases** |
| --- | --- |
| Very low clinical concern for IE | 3 |
| Recurrent case with previously negative TTE | 1 |
| Patient too clinically unstable for TTE to be done | 1 |
| Concurrent SARS-CoV-2 infection | 1 |
| Unclear | 1 |
| **TOTAL** | **7** |

Abbreviations: ID = infectious disease, TTE = transthoracic echocardiography, SARS-CoV-2 = Severe acute respiratory syndrome coronavirus 2, IE = infective endocarditis

##### **Supplementary Table 2**. Secondary outcomes

| **Outcome** | **Number of cases (percentage)** | | | | |
| --- | --- | --- | --- | --- | --- |
|  | **All Cases****(N = 221)** | **TEE performed****(N = 46)** | **TEE not performed****(N = 175)** | **Endocarditis****(N = 39)** | **Not endocarditis****(N = 182)** |
| 30-day all-cause mortality | 14 (6) | 1 (2) | 13 (7) | 6 (15) | 8 (4)* |
| 1-year all-cause mortality | 55 (25) | 9 (20) | 46 (26) | 14 (36) | 41 (23) |
| Recurrent SAB within 1 year | 19 (9) | 5 (11) | 14 (8) | 3 (8) | 16 (9) |
| CDI within 3 months of treatment | 12 (5) | 4 (9) | 8 (5) | 2 (5) | 10 (5) |
| Antibiotic-associated nephrotoxicity | 11 (5) | 1 (2) | 10 (6) | 1 (3) | 10 (5) |
| Antibiotic-associated neutropenia | 3 (1) | 2 (4) | 1 (1) | 1 (3) | 2 (1) |
| Antibiotic-associated thrombocytopenia | 2 (1) | 0 (0) | 2 (1) | 0 (0) | 2 (1) |
| Antibiotic hypersensitivity | 6 (3) | 2 (4) | 4 (2) | 3 (8) | 3 (2) |

##### TTE = transthoracic echocardiography, TEE = transesophageal echocardiography, SAB = *S. aureus* bacteremia, CDI = *C. difficile* infection

##### Groups were compared by Fisher exact test. No outcomes had significant (α = 0.05) differences between the groups.
